# Presence of urinary symptoms in bacteremic urinary tract infection: a retrospective cohort study of *Escherichia coli* bacteremia

**DOI:** 10.1186/s12879-020-05499-1

**Published:** 2020-10-20

**Authors:** Anthony D. Bai, Michael J. Bonares, Samuel Thrall, Chaim M. Bell, Andrew M. Morris

**Affiliations:** 1 Division of Infectious Diseases, McMaster University, 699 Concession St., Hamilton, ON L8N 4A6 Canada; 2 Division of Palliative Medicine, University of Toronto, Toronto General Hospital, 200 Elizabeth St., Toronto, ON M5E 2C4 Canada; 3 Division of Geriatric Medicine, McMaster University, St. Peter’s Hospital Centre for Healthy Aging, 88 Maplewood Ave, Hamilton, ON L8M 1W9 Canada; 4 Antimicrobial Stewardship Program, Sinai Health/University Health Network, Suite 435, 600 University Avenue, Toronto, ON M5G 1X5 Canada; 5 Division of Internal Medicine, Sinai Health, Suite L2-404, 60 Murray Street, Toronto, ON M5T 3L9 Canada; 6 Department of Medicine, University of Toronto, Suite RFE 3-805 200 Elizabeth St, Toronto, ON M5G 2C4 Canada

**Keywords:** *Escherichia coli*, Bacteremia, Urinary tract infection, Diagnosis

## Abstract

**Background:**

It is important to understand clinical features of bacteremic urinary tract infection (bUTI), because bUTI is a serious infection that requires prompt diagnosis and antibiotic therapy. *Escherichia coli* is the most common and important uropathogen. The objective of our study was to characterize the clinical presentation of *E coli* bUTI.

**Methods:**

Retrospective cohort study of consecutive adult patients admitted for community acquired *E. coli* bacteremia from January 1, 2015 to December 31, 2016 was conducted at 4 acute care academic and community hospitals in Toronto, Ontario, Canada. Logistic regression models were developed to identify *E coli* bUTI cases without urinary symptoms.

**Results:**

Of 462 patients with *E. coli* bacteremia, 284 (61.5%) patients had a urinary source. Of these 284 patients, 161 (56.7%) had urinary symptoms. In a multivariable model, bUTI without urinary symptoms were associated with older age (age < 65 years as reference, age 65–74 years had OR of 2.13 95% CI 0.99–4.59 *p* = 0.0523; age 75–84 years had OR of 1.80 95% CI 0.91–3.57 *p* = 0.0914; age > =85 years had OR of 2.95 95% CI 1.44–6.18 *p* = 0.0036) and delirium (OR of 2.12 95% CI 1.13–4.03 *p* = 0.0207). Sepsis by SIRS criteria was present in 274 (96.5%) of all bUTI cases and 119 (96.8%) of bUTI cases without urinary symptoms.

**Conclusion:**

The majority of patients with *E. coli* bacteremia had a urinary source. A significant proportion of bUTI cases had no urinary symptoms elicited on history. Elderly and delirious patients were more likely to have bUTI without urinary symptoms. In elderly and delirious patients with sepsis by SIRS criteria but without a clear infectious source, clinicians should suspect, investigate, and treat for bUTI.

**Supplementary information:**

**Supplementary information** accompanies this paper at 10.1186/s12879-020-05499-1.

## Background

Expert consensus and stewardship interventions emphasize treatment of bacteriuria based on urinary symptoms [1–4]. However, clinicians often diagnose and treat elderly patients for urinary tract infection (UTI) when they have only non-specific symptoms such as delirium based on the belief that elderly patients with UTI may present without localizing symptoms [5–7]. This raises uncertainty as to what constitutes symptoms of UTI, what is asymptomatic bacteriuria, and whether it warrants treatment. Hereafter, we use the term asymptomatic bacteriuria to signify bacteriuria without any urinary symptoms.

Bacteriuria with bacteremia is a true infection requiring treatment, so it can be used to guide diagnostic criteria for UTI. Diagnostic criteria for UTI should capture all bacteremic UTI (bUTI), because it is associated with a higher mortality rate [8, 9]. Prior studies suggested that elderly patients with bUTI often do not have urinary symptoms [10–12], which is recognized by Centre of Disease Control as “asymptomatic bacteremic urinary tract infection” [13]. However, prior studies do not provide alternative clinical features to reliably capture bUTI [10–12].

Approximately 20–30% of patients presenting to emergency department with febrile or complicated UTI or pyelonephritis have bacteremia [14–16]. *Escherichia coli* is the most important pathogen for UTI and accounts for over 70% of all cases [17, 18]. In patients admitted to hospital with *E coli* bacteriuria who had a blood culture done, approximately 15% of patients had *E coli* bacteremia [19].

We conducted a study on *E. coli* bUTI patients to characterize the proportion of and risk factors for bUTI without urinary symptoms. We also aimed to find clinical features that would be sensitive enough to capture bUTI cases without urinary symptoms.

## Methods

### Study design

We conducted a retrospective cohort study at 4 acute care academic and community hospitals in the Greater Toronto Area. Research ethics board approval was obtained from each institution.

The study included consecutive adult patients admitted to the hospital for community acquired *E. coli* bacteremia from January 1, 2015 to December 31, 2016. Community acquired bacteremia was defined by positive blood culture collected at admission or within 48 h of hospital admission. *E. coli* bacteremia was defined as at least 1 positive blood culture for *E. coli*. Patients were excluded if they had an unclear infectious focus. This group was likely composed of patients with urinary source as well as patients with non-urinary source. Analysis and comparison to this mixed patient population would be difficult to interpret.

### Data collection

Data were obtained from electronic and paper medical records at each hospital site and entered into a standardized case report form. Data on demographics, comorbidities, clinical presentation, investigations, microbiological data, investigations, surgical interventions, antibiotic therapy and clinical outcomes were collected. A second auditor performed sample reliability checks on 10% of the population.

### Variable definitions

Comorbidities were entered as per Charlson comorbidity index [20].

Urinary source (i.e. bUTI) required a urine culture with significant monomicrobial growth of *E. coli* > =10 × 10^6 CFU/L and any of the following criteria:

1. Clinical urinary tract infection as per diagnostic criteria for treatment by Loeb et al. [21]. Patients were diagnosed with UTI based on dysuria or > =2 of the following: fever, urgency, flank pain, urinary incontinence, shaking chills, frequency, gross hematuria or suprapubic pain [21]. Patients with urinary catheter were diagnosed with UTI based on > = 1 of the following: new costovertebral tenderness, rigors, new onset delirium or fever [21].
2. Imaging findings suggestive of pyelonephritis including perinephric stranding or hydronephrosis with flank pain or costovertebral angle (CVA) tenderness
3. Recent urologic procedure (including ureteric stenting, cystoscopy, prostate biopsy) with no other clear source
4. Monomicrobial growth of *E. coli* in blood and urine culture with the same susceptibility pattern and no other obvious source on clinical assessment

Biliary source was defined as any of the following:

1. Evidence of cholecystitis, choledocholithiasis, or cholangitis on imaging
2. Known cholecystostomy tube or biliary malignancy with no other obvious source
3. Recent manipulation of biliary tree including ERCP with no other obvious source

Intra-abdominal source was defined as any of the following:

1. Evidence of intra-abdominal abscess, appendicitis, diverticulitis, pancreatitis or mass on abdominal imaging
2. Known intra-abdominal drain with no other obvious source
3. Recent intra-abdominal surgery with no other obvious source

The above criteria were also checked for agreement with the main responsible physician’s diagnosis for accuracy. Other infectious foci including pneumonia were based on the clinician’s final diagnosis.

Urinary symptoms and signs were based on the clinician’s documentation on presentation. Urinary symptoms included dysuria, urinary urgency, urinary frequency, gross hematuria, flank pain, suprapubic pain and urinary retention. Urinary signs included suprapubic tenderness and costovertebral or flank tenderness. Hereafter, bUTI without urinary symptoms refer to bUTI without any of the aforementioned urinary symptoms or signs.

Patients were screened regularly by nurses and assessed by physicians for delirium based on the Confusion Assessment Method (CAM) criteria [22]. Fever was defined as > 37.8C in elderly patients age > 65 [23] and > =38C in all other patients. Sepsis as per the SIRS [24] and qSOFA [25] score were calculated for all patients.

Urinalysis was done using urine test strips.

History of prior UTI was based on patient reporting, as documented in the patient chart by the main responsible physician.

### Outcomes

Patients were followed until death in hospital or discharge. Length of stay was calculated from time of blood culture collection to discharge or death in hospital.

### Statistical analysis

Comparisons between two groups were done with Wilcoxon rank-sum test for non-normally distributed continuous variables and Fisher’s exact test for categorical variables.

Diagnostic properties were determined for clinical factors as the test and urinary source as criterion standard. For example, if dysuria was the test, then a true positive was a patient who had dysuria and bUTI. A true negative was a patient who did not have dysuria and had a non-urinary source for the *E. coli* bacteremia. A false positive was a patient who had dysuria and a non-urinary source for the *E. coli* bacteremia. A false negative was a patient who did not have dysuria, but had bUTI. We calculated sensitivity and specificity with 95% confidence intervals (CI) using the Wilson method. For likelihood ratios, we calculated the 95% CI according to the method described by Simel et al. [26].

In patients with bUTI, a univariate logistic regression model was done predicting bUTI without urinary symptoms. Potential predictors were selected a priori, which included age, gender, stroke, dementia, urinary risk factors, delirium, and severity of infection. Significant predictors were selected based on *p* < 0.2 from univariate analyses. A final multivariable logistic regression model of significant predictors was selected based on clinical judgment, *p*-value, full model with all predictors, as well as both forward and backward stepwise regression based on Akaike information criterion. Hospital site was forced as a predictor into this model.

There are < 5% missing data for all variables, so listwise deletion was done for analyses such as modeling.

All reported CI were 2-sided 95% intervals and all tests were 2-sided with a *P* < 0.05 significance level. All analyses were done with R version 3.6.3 (R Foundation for Statistical Computing, Vienna, Austria).

## Results

**Fig. 1:**
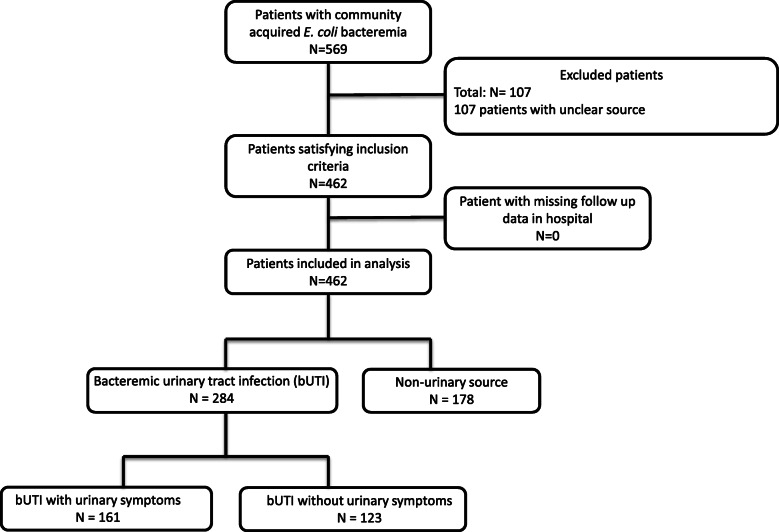
Flow diagram of patients included in this study

In total, 462 patients with *E. coli* bacteremia and a known infectious source were included in the analysis (Fig. 1). Of the 462 patients, 284 (61.5%) patients had a bUTI and 178 (38.5%) patients had a non-urinary source (Table 1). Baseline characteristics of patients with *E coli* bacteremia and an unclear source are described in Additional file 1: Table S1.

**Table 1:** Baseline characteristics of *E. coli* bacteremic patients with bUTI versus non-urinary source

	Patients with bUTI (*N* = 284)	Patients with non-urinary source (*N* = 178)	*p*-value
Demographics			
Age categories			0.3666
< 65 years	85 (29.9%)	41 (23.0%)	
65–74 years	48 (16.9%)	34 (19.1%)	
75–84 years	80 (28.2%)	50 (28.1%)	
> =85 years	71 (25.0%)	53 (29.8%)	
Age median (IQR)	76.0 (62.0–84.7)	77.9 (66.7–86.0)	0.0408
Female	207 (72.9%)	86 (48.3%)	< 0.0001
Long term care home	44 (15.5%)	16 (9.0%)	0.0469
Admitting service			0.0018
Medicine	264 (93.0%)	147 (82.6%)	
Surgery	18 (6.3%)	27 (15.2%)	
ICU	2 (0.7%)	4 (2.3%)	
Charlson comorbidity score			0.0875
0	181 (63.7%)	96 (53.9%)	
1	39 (13.7%)	27 (15.2%)	
> =2	64 (22.5%)	55 (30.9%)	
Charlson comorbidity			
Stroke	21 (7.4%)	8 (4.5%)	0.2416
Dementia	17 (6.0%)	10 (5.6%)	> 0.9999
Diabetes	22 (7.8%)	21 (11.8%)	0.1874
Diabetes with complications	16 (5.6%)	15 (8.4%)	0.2562
Source of infection			
Urinary	284 (0%)	0 (0%)	
Abdomen	0 (0%)	109 (61.2%)	
Biliary	0 (0%)	60 (33.7%)	
Pneumonia	0 (0%)	9 (5.1%)	
History of urinary risk factors			
Chronic indwelling Foley catheter	12 (4.2%)	4 (2.3%)	0.3059
Benign prostate hypertrophy	16 (5.6%)	17 (9.6%)	0.1373
Urinary malignancy	13 (4.6%)	6 (3.4%)	0.6341
Prior urinary tract infection	65 (22.9%)	15 (8.4%)	< 0.0001
Nephrolithiasis	16 (5.6%)	14 (7.9%)	0.3405
Cystoscopy	8 (2.8%)	0 (0%)	0.0259
Prostate biopsy	6 (2.1%)	0 (0%)	0.0875
Other urologic procedure	37 (13.0%)	13 (7.3%)	0.0645
Urinary symptoms			
Dysuria	63 (22.2%)	13 (7.3%)	< 0.0001
Urinary urgency	19 (6.7%)	5 (2.8%)	0.0845
Urinary frequency	61 (21.5%)	5 (2.8%)	< 0.0001
Gross hematuria	14 (4.9%)	2 (1.1%)	0.0351
Flank pain	52 (18.3%)	11 (6.2%)	0.0001
Suprapubic abdominal pain	43 (15.1%)	8 (4.5%)	0.0004
Urinary retention	11 (3.9%)	5 (2.8%)	0.6112
Urinary signs			
Suprapubic tenderness	20 (7.0%)	8 (4.5%)	0.3193
CVA tenderness	28 (9.9%)	6 (3.4%)	0.0098
Any urinary symptoms or signs	161 (56.7%)	41 (23.0%)	< 0.0001
Urinalysis			
Proteinuria	203 / 249 (81.5%)	99 / 139 (71.2%)	0.0220
Hematuria	221 / 249 (88.8%)	101 / 139 (72.7%)	0.0001
Leukocytes	223 / 249 (89.6%)	73 / 139 (52.5%)	< 0.0001
Nitrite	121 / 249 (48.6%)	40 / 139 (28.8%)	0.0002
Leukocytes or nitrites	230 / 249 (92.4%)	79 / 139 (56.8%)	< 0.0001
Severity of Infection			
Delirium	68 (23.9%)	38 (21.4%)	0.5703
Fever	209 (73.6%)	127 (71.4%)	0.5937
Sepsis by SIRS criteria	274 (96.5%)	171 (96.1%)	0.8050
Sepsis by qSOFA criteria	110 (38.7%)	88 (49.4%)	0.0264
Hypotensive shock SBP < 90	223 (78.5%)	136 (76.4%)	0.6462
Transfer to ICU in 72 h	19 (6.7%)	21 (11.8%)	0.0630
Outcome			
Death in hospital	17 (6.0%)	12 (6.7%)	0.8442

Urinary frequency had the highest positive likelihood ratio (PLR) of 7.6 (95% CI 3.1–18.7) for ruling in urinary source (Table 2). Other urinary symptoms and signs have PLR ranging from 1.4 to 4.4 and negative likelihood ratio (NLR) ranging from 0.81 to 0.99. On clinical assessment, no urinary symptoms and signs had a NLR of 0.56 (95% CI 0.48–0.66). Negative leukocytes and nitrites on urinalysis had the lowest NLR of 0.18 (95% CI 0.11–0.28) for ruling out a urinary source.

**Table 2:** Diagnostic utility of symptoms and signs for bacteremic UTI

	Sensitivity % (95% CI)	Specificity % (95% CI)	PLR (95% CI)	NLR (95% CI)
Urinary symptoms				
Dysuria	22 (18–27)	93 (88–96)	3.0 (1.7–5.4)	0.84 (0.78–0.90)
Urinary urgency	7 (4–10)	97 (94–99)	2.4 (0.9–6.3)	0.96 (0.92–1.00)
Urinary frequency	22 (17–27)	97 (94–99)	7.6 (3.1–18.7)	0.81 (0.76–0.86)
Gross hematuria	5 (3–8)	99 (96–100)	4.4 (1.0–19.1)	0.96 (0.93–0.99)
Flank pain	18 (14–23)	94 (89–97)	3.0 (1.6–5.5)	0.87 (0.81–0.93)
Suprapubic abdominal pain	15 (11–20)	96 (91–98)	3.4 (1.6–7.0)	0.89 (0.83–0.94)
Urinary retention	4 (2–7)	97 (94–99)	1.4 (0.5–3.9)	0.99 (0.96–1.02)
Urinary signs				
Suprapubic tenderness	7 (5–11)	96 (91–98)	1.6 (0.7–3.5)	0.97 (0.93–1.02)
CVA tenderness	10 (7–14)	97 (93–98)	2.9 (1.2–6.9)	0.93 (0.89–0.98)
Any urinary symptoms or signs	57 (51–62)	77 (70–83)	2.5 (1.8–3.3)	0.56 (0.48–0.66)
Urinalysis				
Proteinuria	82 (76–86)	29 (22–37)	1.1 (1.0–1.3)	0.64 (0.44–0.93)
Hematuria	89 (84–92)	27 (21–35)	1.2 (1.1–1.4)	0.41 (0.26–0.64)
Leukocytes	90 (85–93)	48 (39–56)	1.7 (1.4–2.0)	0.22 (0.15–0.33)
Nitrite	49 (43–55)	71 (63–78)	1.7 (1.3–2.3)	0.72 (0.62–0.85)
Leukocytes or nitrites	92 (88–95)	43 (35–52)	1.6 (1.4–1.9)	0.18 (0.11–0.28)

Of the 284 patients with bUTI, 123 (43.3%) had no urinary symptoms (Table 3). Fever and/or urinary symptoms were present in 244 (85.9%) patients with bUTI. In the univariate analysis, potential significant predictors of bUTI without urinary symptoms included age, dementia, benign prostate hypertrophy, prior UTI, nephrolithiasis, delirium, sepsis by qSOFA criteria, and hypotensive shock (Additional file 1: Table S2). In the final multivariable model, significant predictors included only age, delirium and prior UTI (Table 4, Additional file 1: Table S3). With increasing age, the proportion of patients with delirium and bUTI without urinary symptoms also increased (Additional file 1: Table S4).

**Table 3:** Characteristics and outcomes of *E. coli* bUTI patients with and without urinary symptoms or signs

	bUTI with urinary symptoms (*N* = 161)	bUTI without urinary symptoms (*N* = 123)	*p*-value
Demographics			
Age categories			0.0025
< 65 years	61 (37.9%)	24 (19.5%)	
65–74 years	26 (16.2%)	22 (17.9%)	
75–84 years	44 (27.3%)	36 (29.3%)	
> =85 years	30 (18.6%)	41 (33.3%)	
Age median (IQR)	72.8 (56.0–81.9)	79.0 (67.5–86.6)	0.0002
Female	122 (75.8%)	85 (69.1%)	0.2271
Long term care home	16 (9.9%)	28 (22.8%)	0.0045
Admitting service			0.4445
Medicine	147 (91.3%)	117 (95.1%)	
Surgery	12 (7.5%)	6 (4.9%)	
ICU	2 (1.2%)	0 (0%)	
Charlson comorbidity score			0.1632
0	96 (59.6%)	85 (69.1%)	
1	27 (16.8%)	12 (9.8%)	
> =2	38 (23.6%)	26 (21.1%)	
Charlson comorbidity			
Stroke	10 (6.2%)	11 (8.9%)	0.4933
Dementia	7 (4.4%)	10 (8.1%)	0.2121
Diabetes	15 (9.3%)	7 (5.7%)	0.3706
Diabetes with complications	13 (8.1%)	3 (2.4%)	0.0660
History of urinary risk factors			
Chronic indwelling Foley catheter	7 (5.7%)	5 (3.1%)	0.3745
Benign prostate hypertrophy	5 (3.1%)	11 (8.9%)	0.0401
Urinary malignancy	9 (5.6%)	4 (3.3%)	0.4041
Prior urinary tract infection	42 (26.1%)	23 (18.7%)	0.1559
Nephrolithiasis	14 (8.7%)	2 (1.6%)	0.0097
Cystoscopy	6 (3.7%)	2 (1.6%)	0.4727
Prostate biopsy	2 (1.2%)	4 (3.3%)	0.4083
Other urologic procedure	24 (14.9%)	13 (10.6%)	0.3739
Severity of Infection			
Delirium	26 (16.2%)	42 (34.2%)	0.0007
Sepsis by SIRS criteria	155 (96.3%)	119 (96.8%)	> 0.9999
Sepsis by qSOFA criteria	53 (32.9%)	57 (46.3%)	0.0268
Hypotensive shock SBP < 90	120 (74.5%)	103 (83.7%)	0.0797
Transfer to ICU in 72 h	9 (5.6%)	10 (8.1%)	0.4746
Empiric antibiotics before blood culture collection	15 (9.3%)	9 (7.3%)	0.6683
Outcome			
Death in hospital	5 (3.1%)	12 (9.8%)	0.0234

**Table 4:** Multivariable logistic regression model predicting bUTI without urinary symptoms

Predictor	Odds Ratio (OR) (95% CI)	*p*-value
Age categories		
< 65 years	Reference	
65–74 years	2.13 (0.99–4.59)	0.0523
75–84 years	1.80 (0.91–3.57)	0.0914
> =85 years	2.95 (1.44–6.18)	0.0036
Delirium	2.12 (1.13–4.03)	0.0207
Prior urinary tract infection	0.56 (0.29–1.04)	0.0699

Hospital site was also forced as a predictor into this model. The estimates for different sites are not shown in this table, but can be found in Additional file 1: Table S3

## Discussion

This retrospective cohort study of patients with *E. coli* bacteremia showed that the majority of patients (61.5%) had a urinary source. A significant proportion of patients (43.3%) with bUTI did not have any urinary symptoms or signs elicited on history or physical exam especially in context of older age and delirium. Even the inclusion of fever and/or urinary symptoms would miss approximately 1 in 7 bUTIs. Sepsis by SIRS criteria was present in almost all of the bUTI cases without urinary symptoms.

We used a comparison group of non-urinary source *E. coli* bacteremia patients. The majority of these patients had intra-abdominal or hepato-biliary infection, similar to prior studies [27, 28]. Patients with a non-urinary source had similar outcomes including mortality compared to patients with urinary source. We believe this is an appropriate comparison group as it simulates a common scenario whereby a patient presents with sepsis or *E. coli* bacteremia of unknown source, and the clinician must evaluate and determine the source of infection.

Our finding of a significant proportion of bUTI presenting without urinary symptoms in elderly patients is consistent with prior studies on bUTI [11, 12] and non-bacteremic UTI [29]. Although our study found urinary frequency to be useful for ruling in UTI, it demonstrated little diagnostic utility in a systematic review of observational studies [29]. This may be due to the wide variation in the case definition of UTI that includes asymptomatic bacteriuria without true infection. The usefulness of urinalysis in ruling out UTI in our study was similarly found in prior studies with a reported high sensitivity [30] and negative predictive value [31].

There are several reasons why an elderly patient with delirium and a UTI may have no urinary symptoms. Frail adults may have atypical presentation of infections [6, 32]. Even if such patients experience UTI symptoms, they may not be able to describe or demonstrate them due to cognitive dysfunction [6]. While clinical evaluation for typical urinary symptoms and signs were not sensitive to capture all bUTI, sepsis by SIRS criteria came close. The qSOFA components such as altered mental status and tachypnea tend to be non-specific for infection in elderly patients [33, 34]. This may lead to overtreatment and missed diagnosis of delirium causes other than infection [8]. In our study, qSOFA was not sensitive for bUTI, being positive in only 38.7% of all bUTI patients and 46.3% of bUTI patients without urinary symptoms. In contrast, SIRS criteria have been shown previously to be sensitive in identifying bacteremia in all patients [35] including elderly patients in particular [33]. Therefore, physicians may continue to use SIRS criteria for decisions on when to draw blood cultures, especially in elderly patients.

This study has several strengths. It included a large population of 284 bUTI cases compared to prior studies that ranged from 61 to 191 patients [10–12]. It was conducted across academic and community hospitals, which increases its generalizability. Also, by limiting inclusion to those patients with bacteremia, it allows characterization of UTI without urinary symptoms and largely eliminates the inclusion of asymptomatic bacteriuria without true infection.

Our study has limitations that merit discussion. First, there are the inherent limitations from a retrospective chart review. However, the data collection was rigorous and a second auditor had performed sample data checking to ensure completeness and quality. The clinical assessment of patients for urinary symptoms and signs were not standardized and may vary between clinicians. Almost all patients in our study had sepsis as per SIRS or qSOFA criteria, and had a blood culture drawn at presentation. Therefore, all patients should have undergone a systematic evaluation for possible infectious foci including UTI. As well, the non-standardized approach reflects real world settings. Second, there may be ascertainment bias. The study could only capture patients in whom blood cultures were drawn. Physicians may be more likely to order blood cultures for patients in whom the symptoms are non-specific or the infectious source is uncertain. The study also likely selected sicker patients with higher suspicion for bacteremia. This could lead to overestimation of the diagnostic properties but does not affect our most important findings: that UTI is the most common cause of *E. coli* bacteremia, and most elderly or delirious patients had bUTI without urinary symptoms. Third, our study captured bUTI by *E. coli* only, so it would be an extrapolation to all bUTI. Nonetheless, *E coli* is the most common and important pathogen responsible for approximately 70% of pyelonephritis [17]. Fourth, UTI presentation may be different and unique in patients with spinal cord injury or disease. Unfortunately, our study did not collect information on spinal cord injury or disease. It is likely that many of these patients would have been captured in the variable of chronic indwelling Foley catheter.

These findings offer insight in the clinical presentation of *E coli* bUTI. Urinary symptoms are useful to diagnose UTI. However, elderly and/or delirious patients may have a bUTI despite having no urinary symptoms or signs elicited on history or exam. For these cases, in addition to symptoms and signs, SIRS criteria and positive urinalysis without any other clear infectious source may be important clues to a bUTI that require antibiotic therapy. Our study should be interpreted with caution and not be extrapolated to *E coli* bacteriuria without bacteremia. This is outside the scope of our study. Thus, our study does not address which patients are at risk for bUTI. This should be explored in future studies.

Our study adds to the evidence that UTI without urinary symptoms is common and important in elderly and/or delirious patients. We hope that this study can contribute towards a meaningful update of the concept of UTI symptoms. Emphasis on urinary symptoms without consideration of other aspects of the patient’s presentation is potentially harmful and may miss bUTI. A more holistic approach would consider other clinical factors including age, delirium and sepsis by SIRS criteria. This approach may help increase the chance of antibiotics being given to those who truly need it.

## Conclusions

Most of the patients with *E. coli* bacteremia had a urinary source. A significant proportion of bUTI cases had no urinary symptoms. Elderly and delirious patients were more likely to have bUTI without urinary symptoms. In these elderly and delirious patients who satisfied SIRS criteria but without a clear infectious source, clinicians should suspect, investigate, and treat for bUTI.

## Supplementary information

**Additional file 1: Table S1.** Comparison of baseline characteristics of *E. coli* bacteremic patients with urinary source, non-urinary source and unclear source. **Table S2.** Univariate logistic regression model predicting bUTI without urinary symptoms. **Table S3.** Multivariable logistic regression model predicting bUTI without urinary symptoms. **Table S4.** Proportion of delirium and UTI without urinary symptoms in different age categories in patients with bUTI.

## Data Availability

The data sets used and/or analyzed during the current study are available from the corresponding author on reasonable request.

## Abbreviations

UTI: Urinary tract infection
bUTI: bacteremic urinary tract infection
