# Supplementary material for: Presence of urinary symptoms in bacteremic urinary tract infection: a retrospective cohort study of Escherichia coli bacteremia
Source: BMC Infect Dis. 2020 Oct 20;20:781. doi: 10.1186/s12879-020-05499-1 (PMC7576869; doi:10.1186/s12879-020-05499-1)
Supplement: Supplementary file 1 — Additional file 1: Table S1. Comparison of baseline characteristics of E. coli bacteremic patients with urinary source, non-urinary source and unclear source. Table S2. Univariate logistic regression model predicting bUTI without urinary symptoms. Table S3. Multivariable logistic regression model predicting bUTI without urinary symptoms. Table S4. Proportion of delirium and UTI without urinary symptoms in different age categories in patients with bUTI. [file 12879_2020_5499_MOESM1_ESM.docx]

**Supplementary Material**

Table S1. Comparison of baseline characteristics of *E. coli* bacteremic patients with urinary source, non-urinary source and unclear source

|  | Patients with bUTI  (N=284) | Patients with non-urinary source  (N=178) | Patients with unclear source  (N=107) | p-value |
| --- | --- | --- | --- | --- |
| Demographics |  |  |  |  |
| Age categories |  |  |  | 0.3268 |
| <65 years | 85 (29.9%) | 41 (23.0%) | 27 (25.2%) |  |
| 65 – 74 years | 48 (16.9%) | 34 (19.1%) | 28 (26.2%) |  |
| 75 – 84 years | 80 (28.2%) | 50 (28.1%) | 27 (25.2%) |  |
| >=85 years | 71 (25.0%) | 53 (29.8%) | 25 (23.4%) |  |
| Age median (IQR) | 76.0 (62.0-84.7) | 77.9 (66.7-86.0) | 74.8 (64.7-83.9) | 0.1156 |
| Female | 207 (72.9%) | 86 (48.3%) | 53 (49.5%) | <0.0001 |
| Long term care home | 44 (15.5%) | 16 (9.0%) | 26 (24.3%) | 0.0022 |
| Admitting service |  |  |  | <0.0001 |
| Medicine | 264 (93.0%) | 147 (82.6%) | 0 (0%) |  |
| Surgery | 18 (6.3%) | 27 (15.2%) | 107 (100%) |  |
| ICU | 2 (0.7%) | 4 (2.3%) | 0 (0%) |  |
| Charlson comorbidity score |  |  |  | <0.0001 |
| 0 | 181 (63.7%) | 96 (53.9%) | 98 (91.6%) |  |
| 1 | 39 (13.7%) | 27 (15.2%) | 1 (0.93%) |  |
| >=2 | 64 (22.5%) | 55 (30.9%) | 8 (7.5%) |  |
| Charlson comorbidity |  |  |  |  |
| Stroke | 21 (7.4%) | 8 (4.5%) | 3 (2.8%) | 0.1894 |
| Dementia | 17 (6.0%) | 10 (5.6%) | 4 (3.7%) | 0.7328 |
| Diabetes | 22 (7.8%) | 21 (11.8%) | 5 (4.7%) | 0.1030 |
| Diabetes with complications | 16 (5.6%) | 15 (8.4%) | 0 (0%) | 0.0026 |
| History of urinary risk factors |  |  |  |  |
| Chronic indwelling Foley catheter | 16 (5.6%) | 6 (3.4%) | 3 (2.8%) | 0.5526 |
| Benign prostate hypertrophy | 16 (5.6%) | 17 (9.6%) | 3 (2.8%) | 0.0687 |
| Urinary malignancy | 13 (4.6%) | 6 (3.4%) | 3 (2.8%) | 0.7388 |
| Prior urinary tract infection | 65 (22.9%) | 15 (8.4%) | 16 (15.0%) | 0.0002 |
| Nephrolithiasis | 16 (5.6%) | 14 (7.9%) | 1 (0.9%) | 0.0276 |
| Cystoscopy | 8 (2.8%) | 0 (0%) | 0 (0%) | 0.0155 |
| Prostate biopsy | 6 (2.1%) | 0 (0%) | 0 (0%) | 0.0500 |
| Other urologic procedure | 37 (13.0%) | 13 (7.3%) | 0 (0%) | <0.0001 |
| Urinary symptoms |  |  |  |  |
| Dysuria | 63 (22.2%) | 13 (7.3%) | 13 (12.2%) | <0.0001 |
| Urinary urgency | 19 (6.7%) | 5 (2.8%) | 0 (0%) | 0.0041 |
| Urinary frequency | 61 (21.5%) | 5 (2.8%) | 5 (4.7%) | <0.0001 |
| Gross hematuria | 14 (4.9%) | 2 (1.1%) | 5 (4.7%) | 0.0672 |
| Flank pain | 52 (18.3%) | 11 (6.2%) | 6 (5.6%) | <0.0001 |
| Suprapubic abdominal pain | 43 (15.1%) | 8 (4.5%) | 8 (7.5%) | 0.0006 |
| Urinary retention | 11 (3.9%) | 5 (2.8%) | 0 (0%) | 0.0872 |
| Urinary signs |  |  |  |  |
| Suprapubic tenderness | 20 (7.0%) | 8 (4.5%) | 0 (0%) | 0.0055 |
| CVA tenderness | 28 (9.9%) | 6 (3.4%) | 0 (0%) | <0.0001 |
| Any urinary symptoms or signs | 161 (56.7%) | 41 (23.0%) | 26 (24.3%) | <0.0001 |
| Urinalysis |  |  |  |  |
| Proteinuria | 203 / 249 (81.5%) | 99 / 139 (71.2%) | 62 / 74 (83.8%) | 0.0346 |
| Hematuria | 221 / 249 (88.8%) | 101 / 139 (72.7%) | 62 / 74 (83.8%) | 0.0004 |
| Leukocytes | 223 / 249 (89.6%) | 73 / 139 (52.5%) | 44 / 74 (59.5%) | <0.0001 |
| Nitrite | 121 / 249 (48.6%) | 40 / 139 (28.8%) | 18 / 74 (24.3%) | <0.0001 |
| Leukocytes or nitrites | 230 / 249 (92.4%) | 79 / 139 (56.8%) | 47 / 74 (63.5%) | <0.0001 |
| Severity of Infection |  |  |  |  |
| Delirium | 68 (23.9%) | 38 (21.4%) | 26 (24.3%) | 0.7747 |
| Fever | 209 (73.6%) | 127 (71.4%) | 73 (68.2%) | 0.5558 |
| Sepsis by SIRS criteria | 274 (96.5%) | 171 (96.1%) | 95 (88.8%) | 0.0129 |
| Sepsis by qSOFA criteria | 110 (38.7%) | 88 (49.4%) | 42 (39.3%) | 0.0628 |
| Hypotensive shock SBP <90 | 223 (78.5%) | 136 (76.4%) | 77 (72.0%) | 0.3748 |
| Transfer to ICU in 72 hours | 19 (6.7%) | 21 (11.8%) | 1 (0.9%) | 0.0011 |
| Outcome |  |  |  |  |
| Death in hospital | 17 (6.0%) | 12 (6.7%) | 7 (6.5%) | 0.9219 |

Table S2. Univariate logistic regression model predicting bUTI without urinary symptoms

| Predictor | Odds Ratio (OR)  (95% CI) | p-value |
| --- | --- | --- |
| Demographics |  |  |
| Age categories |  |  |
| <65 years | Reference |  |
| 65 – 74 years | 2.15 (1.03-4.53) | 0.0421 |
| 75 – 84 years | 2.08 (1.10-4.00) | 0.0263 |
| >=85 years | 3.47 (1.80-6.85) | 0.0003 |
| Male | 1.40 (0.83-2.37) | 0.2111 |
| Charlson comorbidity |  |  |
| Stroke | 1.48 (0.60-3.68) | 0.3858 |
| Dementia | 1.95 (0.73-5.51) | 0.1898 |
| History of urinary risk factors |  |  |
| Chronic indwelling Foley catheter | 1.88 (0.59-6.50) | 0.2902 |
| Benign prostate hypertrophy | 3.06 (1.08-9.95) | 0.0430 |
| Urinary malignancy | 0.57 (0.15-1.79) | 0.3559 |
| Prior urinary tract infection | 0.65 (0.36-1.15) | 0.1440 |
| Nephrolithiasis | 0.17 (0.03-0.64) | 0.0222 |
| Cystoscopy | 0.43 (0.06-1.89) | 0.3026 |
| Prostate biopsy | 2.67 (0.51-19.51) | 0.2610 |
| Other urologic procedure | 0.67 (0.32-1.37) | 0.2840 |
| Severity of infection |  |  |
| Delirium | 2.69 (1.55-4.77) | 0.0005 |
| Sepsis by SIRS criteria | 1.15 (0.32-4.59) | 0.8300 |
| Sepsis by qSOFA criteria | 1.76 (1.09-2.86) | 0.0219 |
| Hypotensive shock SBP <90 | 1.76 (0.98-3.24) | 0.0631 |
| Empiric antibiotics before blood culture collection | 0.77 (0.31-1.79) | 0.5492 |

Table S3. Multivariable logistic regression model predicting bUTI without urinary symptoms

| Predictor | Odds Ratio (OR)  (95% CI) | p-value |
| --- | --- | --- |
| Hospital site |  |  |
| A | Reference |  |
| B | 0.80 (0.37-1.73) | 0.5728 |
| C | 1.35 (0.65-2.85) | 0.4234 |
| D | 1.89 (0.85-4.26) | 0.1199 |
| Age categories |  |  |
| <65 years | Reference |  |
| 65 – 74 years | 2.13 (0.99-4.59) | 0.0523 |
| 75 – 84 years | 1.80 (0.91-3.57) | 0.0914 |
| >=85 years | 2.95 (1.44-6.18) | 0.0036 |
| Delirium | 2.12 (1.13-4.03) | 0.0207 |
| Prior urinary tract infection | 0.56 (0.29-1.04) | 0.0699 |

Table S4. Proportion of delirium and UTI without urinary symptoms in different age categories in patients with bUTI

|  | <65 years  N=85 | 65-74 years  N=48 | 75-84 years  N=80 | >=85 years  N=71 |
| --- | --- | --- | --- | --- |
| UTI without urinary symptoms | 24 (28.2%) | 22 (45.8%) | 36 (45.0%) | 41 (57.8%) |
| Delirium | 6 (7.1%) | 8 (16.7%) | 21 (26.3%) | 33 (46.5%) |
| Fever | 61 (71.8%) | 35 (72.9%) | 59 (73.8%) | 54 (76.1%) |
| Sepsis by SIRS criteria | 83 (97.7%) | 46 (95.8%) | 74 (92.5%) | 71 (100%) |
| Sepsis by qSOFA criteria | 32 (37.7%) | 17 (35.4%) | 28 (35.0%) | 33 (46.5%) |
| Hypotensive shock SBP <90 | 64 (75.3%) | 33 (68.8%) | 64 (80.0%) | 62 (87.3%) |
| Death in hospital | 2 (2.4%) | 3 (6.3%) | 8 (10.0%) | 4 (5.6%) |
